# Influences

**Published:** 1880-06

**Authors:** 


### INFLUENCES.

For weal or woe, how are they falling around our children, especially in large cities, every hour of their existence, and how wide awake should every parental heart be to the direction of the character of those influences! A short time since, one of our daily papers, in noticing the death of an individual, says, "He was a man of undoubted talent, and had he fallen under proper influences, might have achieved a reputation and secured a fortune; as it was, he died at forty-two, without character or morals, a drunkard, an outcast, and a forger."

What were some of the malign influences which shaped this man's course for infamy, who otherwise might have been a credit to the nation and an honor to his kind? The love of dress, the love of drink, and the love of the drama. Foppery, brandy and the theatre were his ruin, as they have been the ruin of countless multitudes before. And what were some of the "*proper influences*" which the notice above intimates would have worked out a different destiny?—the influence of a home, made happy in childhood, by parental unity and piety—by sisterly purity and affection, and by such a remembrance of the sabbath day, as secures it to be spent in the sanctuaries of religion.

